# The Bristol Meeting of the British Medical Association, 1894

**Published:** 1894-09

**Authors:** 


*Psychology.*—The work in this Section was characterised by great activity, and the attendances were larger than on any similar occasion. The meetings were held in Lecture Room, No. 4, University College. On Wednesday, the President, Dr. G. Fielding Blandford, opened the proceedings by delivering a masterly address upon the Prevention of Insanity. The subject is a grand one, and the fullest justice was done to it from all points of view. The most practical part of it dealt with the prevention of marriages between neurotic persons, the limiting of the families of such persons, the appropriate bringing up of children hereditarily predisposed to mental disease, and various other points of mental hygiene. Discussion of the address was specially invited, and an animated debate followed, in which admiration was freely expressed for the courage which the President had shown in being the first man of leading position to advocate openly before a representative assembly some of the views expressed that day, and which were generally held by alienists. A minor point which was debated at some length had reference to the wisdom or otherwise of warning boys and girls about the evils of self-abuse.

Dr. Fletcher Beach and Dr. G. E. Shuttleworth introduced a discussion on Thursday on "The Education of Feeble-Minded Children." Both the contributions took into consideration the Association Report of the Committee on the Examination of School Board Children, which was printed in the *Daily Journal.* Dr. Beach presented a critical analysis of the observations, amongst the most important of which was the group of cases described as "exceptional children." Amongst others, the group included cases of imbeciles, children feebly gifted mentally, children mentally exceptional, epileptics, and dumb children. The defects of development which were combined with each of these classes were described, and it was noted that the mentally exceptional children were those who, with fair intelligence, were of feeble or low moral character. The truants, excitable children, and those of irregular mental conformation were examples of this class, who, if not specially trained, committed late in life some offence, and were afterwards numbered among the criminal population. The facts observed were summarised, and the means to be adopted for the improvement of those who were mentally dull or mentally exceptional were pointed out. Dr. Shuttleworth offered some practical observations on the useful hints furnished by the classification adopted in the Report. He urged that special modes of education adapted to special defects were called for in the case of children incapacitated by nervous or mental defects from following the ordinary school curriculum; that special training was necessary for the teacher, beyond that of the Kindergarten, enabling her to recognise the physical signs of mental abnormality before harm was done by pushing instruction where it could not be tolerated, and to direct her efforts intelligently so as to counteract incapacities and develop mental power through the education of the senses. "The Law in Relation to the Criminal Responsibility of the Insane" was the next subject for discussion. It was admirably opened by Dr. Lionel Weatherly, who showed how variously the law is interpreted by different judges when the plea of insanity is raised in criminal cases. He clearly demonstrated that either the law requires alteration or the practice of the judges should be assimilated. In the lively debate which followed, Mr. Pitt Lewis, Q.C., considered that no alteration of the law was required, provided that all the judges would interpret it in the wide and liberal sense which some of them had already adopted; and Mr. Ernest Hart promised the help of the Parliamentary Bills Committee of the Association in accomplishing the desirable reform of the existing condition of things. Altogether this discussion was the chief feature of the Sectional work, and resolutions to take practical steps to put an end to the present anomalous position were passed unanimously and with enthusiasm. In the afternoon a number of members, led by the President and other officers of the Section, availed themselves of a kind invitation to visit the Bristol City and County Asylum, where they were received by Dr. Benham, the Superintendent, and by the Chairman of the Committee. Even the most experienced of the visitors must have learned something, and all of them expressed in laudatory terms their gratification with what they saw.

The early part of Friday was devoted to lantern demonstrations of certain changes in nerve cells and of improvement effected in cases of sporadic cretinism by treatment with thyroid extract.

*Pathology.*—On the first morning in the Pathological Section, which was held in the Chemical Lecture Theatre of University College, Dr. S. Monckton Copeman introduced a discussion on "The Pathology of Vaccinia." At the Nottingham meeting the protozoa of cancer were the objects of interest. At Bristol the protozoa of vaccinia occupied the attention of the Section. But the discussion tended, as was foreseen, to drift away from the pathology of vaccinia to allied topics, and one member complained that he wanted to hear about that, and not to listen to a discussion on questions with reference to vaccination. However, the Section was not stormed by anti-vaccinators, and peace reigned throughout the debate. Those whose minds had a surgical tendency were refreshed towards the end of the morning by a demonstration by Mr. Targett, Curator of the College of Surgeons of England Museum, of very beautiful lantern slides illustrating hydatid disease of bone.

On the following morning there was a series of papers on lesions of the spinal cord. Some of them were illustrated by lantern slides, which showed the tracts of degeneration in a very striking way.

On Friday, papers on various topics of pathology were read.

Some reform is needed in connection with this Section. Pathology is not a separate science. The physician, the surgeon and the specialist have each a separate pathology. But they each have a sectional meeting, and to that they naturally go. The result is that about a dozen, who devote themselves to bacteriology or chemical pathology, are left to attend the Pathological Section and read papers to one another. Outside these there is practically no audience at all. It would be better if papers dealing with pathology were read in the respective sections to which they more particularly belong, and where interested listeners would be already assembled. If these papers must be read in a special pathology section, then this should be when the other sections are not sitting; but, under existing arrangements, it is difficult to see when this could be done. Possibly conjoint meetings with some other sections, perhaps Medicine or Surgery or both, might be arranged.

*Ophthalmology.*—The meetings of this Section were held in the Medical Lecture Theatre of the School. After a few introductory remarks the President, Mr. F. R. Cross called upon Mr. Simeon Snell to read his paper on "The Relations of Occupation to Eyesight," a subject on which information had been especially invited. Injury to sight from occupations involving exposure to light, heat, dust, and poisonous gases was dealt with, and suggestions were made for preventing ill effects. This was followed by a paper by Dr. Lindsay Johnson, who had made special study of the well-known failure of vision of the men employed in Salviati's Venetian Glass Works. His investigations led him to the conclusion that the partial optic atrophy to which the amblyopia was due resulted from the bright light from the furnace, and was not in any way attributable to the heat. Mr. R. W. Doyne spoke of the influence of education on eyesight, the ill-effects of which he thought were exaggerated. He especially insisted on the harm often done by the injudicious use of blue and neutral-tint glasses. In the discussion which followed allusion was made to various trades which are injurious to the eyes. Mr. Ernest Clarke brought forward a resolution to the effect that it was desirable that employers of labour should be compelled to see that their men were provided with *and used* proper precautions for preventing injury to their sight. It was, however, decided that, though right in principle, the proposition was premature. Prof. Landolt detailed some rules which he used for determining the affected muscle in cases of diplopia. He showed a very convenient chart, devised by himself, which indicates at a glance the affected muscle and the position of the false image. Mr. Risien Russell gave a very clear and interesting account of a series of experiments which he had performed on monkeys with a view to determining the cortical brain-centres for eye-movements. Until he made these experiments only associated lateral movements had been obtained, or, at any rate, they had very largely predominated. He concurred with the view suggested by Dr. Hughlings Jackson that this was due to lateral movements being of first importance to the life of the animal. By means of dividing certain of the ocular muscles and thereby eliminating the possibility of lateral movements, and by using suitable stimuli, Mr. Russell had been able to discover the cortical centres for upward and downward movements of the eyes, and also for convergence.

The second meeting of the Section was commenced by Mr. Juler reading a paper on "The Diagnosis and Treatment of Catarrhal, Purulent, and Granular Ophthalmia," in which he insisted on the use of the microscope in searching for gonococci and trachoma-cocci. Mr. Kenneth Scott advocated a 2 or 4 per cent. solution of cyanide of mercury as a treatment for trachoma more efficacious and less painful than perchloride, which is also a less stable salt than the cyanide. Dr. Hermann Knapp advocated "expression" with his forceps as a valuable auxiliary to other methods. Other speakers stated that sulphate of copper was the best application. Allusion was made to the difficulty of distinguishing true trachoma by microscopic sections of the conjunctiva. Mr. John Griffith read a paper on "The Secretory Function of the Ciliary Body," which was illustrated by excellent drawings and microscopic sections. In one of the latter the radial muscular fibres of the iris (pupil-dilator fibres) were well shown. By means of a modification of the bleaching process introduced by Mr. Treacher Collins two years ago, Mr. Griffith was able to show much evidence that the aqueous humour is secreted by the large ciliary processes and not by the glands of the ciliary region described by Mr. Collins. These latter do not exist in albino rabbits, and therefore Mr. Griffith suggests that they control the amount of the uveal pigment.

Among the papers of the third day was one by Mr. G. E. Walker, who explained a new operation consisting of numerous punctures or incisions through the sclero-corneal junction for cases where Saemisch's section had been hitherto practised.

It was at one time feared that the International Ophthalmological Congress to be held in Edinburgh a week later than the Association meeting would render this section needless, or, at all events, uninteresting. This, however, was not the case for not only were there numerous and important papers, but the attendance was above the average, and on one occasion there were 46 present.

*Laryngology and Otology.*—The work of this Section, the meetings of which were held in the Physiological Lecture Theatre of the School, was opened by a presidential review of the recent advances that had been made in the departments of work coming within the scope of the Section. Of these advances perhaps the least important were in the branch of rhinology, yet the progress in all has been on sound lines, taking as foundations patient clinical research, physiology, pathology, and rational therapeutics. The tendency has been to substitute operative measures for treatment by drugs. And this change has been productive of immense benefit, but much caution is needed in operations which are more or less experimental. The man who experiments in this way must have already gained a high position, the patient must know the experimental nature of the procedure, and its performance must rest upon a basis of perfect logic. The speciality is in danger, too, from excessive attention to unimportant details. A discussion on "The Prognosis of Chronic Non-Suppurative Otitis Media" was opened by Mr. G. P. Field and Dr. Thomas Barr, who treated the subject from somewhat different standpoints. Mr. Field classified cases into favourable and unfavourable, according to the duration of the symptoms, the cause of the malady, the occupation of the patient, the mode of onset, the accompanying symptoms, &c.; while Dr. Barr took a pathological classification as the basis of his prognosis, and divided cases into (1) the exudative form, with swelling and hyper-secretion of the mucous membrane; (2) the non-exudative class, attended by interstitial change with new formation of connective tissue in the mucous membrane; (3) the mixed class, in which the exudative form of inflammation is superadded to an already existing interstitial form; and (4) the class in which these are complicated with a disturbance in the labyrinth. The discussion which followed was as good as could be expected considering the somewhat uninteresting nature of the topic.

The second day was mainly occupied by a discussion on "Diphtheria." On this occasion a conjoint meeting was formed by the union of this Section with that of Diseases of Children. Dr. W. P. Northrup opened the discussion by advocating the claims of two methods in the treatment of true croup and laryngeal diphtheria. If calomel fumigation failed, then recourse should be had to intubation. As he had been closely associated with Dr. O'Dwyer for fourteen years, it was intensely interesting to listen to Dr. Northrup's references to the difficulties encountered by his great master, and to the patience and perseverance with which he had overcome each and every obstacle that he had met with in developing his method. Science, like virtue, is its own reward; and as is too often the case, notwithstanding the immense benefits that have been conferred on humanity, the inventor has received comparatively little pecuniary return for his unselfish labour. Intubation is no doubt an interesting subject, but it hardly covers the whole ground of the projected debate, and as nearly all the available time was taken up in its consideration, there was little opportunity for those who wanted to discuss the diagnosis of diphtheria. Dr. Hutton, who was to have introduced the question in the Children's Section, thought that his paper had better remain unread. Although this combined meeting secured a large audience, it is doubtful if it is wise to bring together two sections for a discussion such as this. Many members of each section were heard to declare that it was unsatisfactory.

The third day was given up to a discussion on "The Diagnosis and Treatment of Empyema of the Nasal Accessory Sinuses." This was introduced by Dr. Greville Macdonald and Mr. Charters Symonds, who confined their attention almost entirely to the frontal and ethmoidal sinuses, saying very little about the better known subject of empyema of the maxillary sinus. Very little time remained for discussion, as the president deemed it his duty to close the meeting of the Section at eleven o'clock punctually, in order to allow members to hear the address in Public Health. Some other presidents of sections did not follow this excellent example, but allowed their discussions to go on for half-an-hour longer, the consequence being that members came straggling into the general address all through its delivery, causing great disturbance.

Some objection was raised this year to the combination of otology and laryngology in one section. It was said that whenever they were combined the result was failure, but that when the subjects were taken singly in alternate years good meetings were obtained. The result at Bristol proves that it is possible to successfully combine the two subjects if a president is chosen who is interested in both. The authorities could not have made a happier selection than Dr. McBride, who is an old Cliftonian. In the conduct of the work of the Section he added great firmness to unvarying courtesy, and thus kept all unruly elements within due bounds.

*Dermatology.*—This Section met in Lecture Room, No. 3, University College. Dr. A. J. Harrison, the President, began the proceedings by an address on "The Germ Theory in Dermatology," pointing out how recent investigations have established more and more the germ sources of many skin affections. Bacteriology, although it has thrown much light upon some obscure phases of dermatology, will not explain all phenomena, and the germ hobby must not be ridden too hard. Common sense must have its leavening power. We want to know what causes the outward manifestations. Dr. Harrison also emphasised the importance of rest in the treatment of skin diseases. A discussion then took place on "Lupus: its Etiology, Pathology, and Relation to other Forms of Cutaneous Tuberculosis." It was introduced in a paper by Prof. Leloir, who was unfortunately absent owing to illness. The writer dealt with the experimental evidence of the tuberculous nature of lupus, which he considers to be a form of tuberculosis of the skin. Subsequent speakers were inclined to support Prof. Leloir's views.

On Thursday morning, Mr. Malcolm Morris opened the discussion on "The Management of Eczema," in a very clear and incisive address. He attaches but little importance to constitutional states, regarding them as only coincidental in such a wide-spread complaint as eczema. He did not regard, however, all cases as microbic. Every case of eczema should be cured as speedily as possible, without fear of producing constitutional troubles. Local remedies and prophylaxis are all important. Mr. Morris's points were well received, except by a few illogical speakers, who were evidently unable to distinguish between true eczema and dermatitis caused by irritants. An interesting discussion was followed by Dr. Unna's very concise demonstration of the micrococcus of eczema, which, by new processes of preparing and staining, can be readily obtained in all cases of true eczema. He has named it "morococcus" on account of its mulberry-like appearance. Two important characteristics of it were pointed out: one is that it is found inside leucocytes, thus differing from the staphylococcus of pus; the other is that it must have oxygen, and therefore is most abundant in the crusts of the disease. Hence, by removing the crusts and preventing their re-formation, and sealing over the pores with suitable remedies, the most successful and scientific kind of treatment can be adopted.

On the third day there was a discussion on "The Nervous Origin of Skin Diseases," which was very ably opened by Dr. Radcliffe Crocker, who, at very short notice, had taken the place of Dr. Pringle, unable to come to the meeting. A table had been prepared with three headings of certain nerve lesions which seemed to be followed by a tendency to certain skin affections. A capital debate followed. Dr. Stephen Mackenzie opened a discussion on "The Etiology and Treatment of Acne." After insisting upon correct views of its pathology, he said that the treatment which is based upon them is the diligent application of hot water to remove superfluous sebum and epithelial accumulations, and then the use of medicated soap to keep the skin aseptic and stimulate the sebaceous glands into healthy activity. After an interesting discussion, Dr. Unna had another bacillus to demonstrate which he had found in acne, but not in ordinary inflammation of the sebaceous follicles. Other good work was done, to some of which attention is called in the Report of the "Progress of the Medical Sciences" in another part of this number.

It is not too much to say that this was probably the best Dermatological Section the Association has ever had. The subjects for debate were wisely chosen. In the discussions on these and on many of the papers there was enthusiasm and continued interest, and the attendance of members was numerous.

*Diseases of Children.*—The meetings of this Section were held in Lecture Room, No. 5, University College. Dr. Howship Dickinson, the President, was unfortunately unable to attend. His opening address was read by Dr. J. E. Shaw. It dealt with "Some of the Characteristics of Disease in Childhood," and pointed out some of the peculiarities of disease in early life, and the difference between the morbid proclivities of youth and age. In the period of growth the processes of nutrition are more active than when the fabric is stationary. Physiology and pathology are so little separated that it is well to remember that frequent nourishment is called for in childhood, and attention to warmth is specially required. The liability to rapid exhaustion from discharge is often overlooked. The most exaggerated examples of cirrhosis are to be found in youth, and sometimes from alcohol. The address then passed in review some aspects of the affections of the respiratory and urinary organs, and concluded by saying that children are more immediately affected by aerial influences, whether good or bad, than are grown people; and this led up to a plea for seaside hospitals and country resorts, rather than the multiplication of town hospitals for children. Dr. Carmichael then opened a discussion on "The Treatment and Complications of Whooping-cough." After dealing with its etiology and its complications, he insisted upon the treatment being based on the fact that it is a zymotic disease, running a certain course, and in which prophylaxis is so important that the affection should be included in the compulsory notification list.

As mentioned in the notice of the Laryngology Section, a combined meeting was held on the second day for the consideration of Diphtheria. Dr. Northrup, who took a considerable part in this, also showed, for Dr. O'Dwyer, an apparatus for artificial respiration, which from the description seemed to be as simple as it was efficacious.

On Friday, the question of "The Treatment of Tuberculous Disease of Joints in Children" was introduced by Mr. Frederic Eve, who said that the subject was one in which the widest divergencies of opinion and of practice exist. But whatever diversities there may be in regard to the general treatment, there can be no doubt about the guiding principle which is, to assist the tissue cells to circumscribe and ultimately to obliterate the disease by rest, extension, and other measures, and to invigorate the cells by good feeding, by placing the patient in the best hygienic conditions, and possibly by the exhibition of certain internal remedies. Various methods of practice, including the novel plan of the artificial induction of venous hyperæmia, were passed in review, and an interesting discussion followed.

Although the work of this Section was of a high order—for the discussions were good and the papers, without exception, interesting and instructive,—there does not seem any real necessity for a Section on Diseases of Children, for the consideration of these ailments might well come in the several departments to which they could scientifically be referred.

There is a very general opinion that, considering the necessities of the social functions, a due observance of which contributes to the scientific success of the meeting, two o'clock is too late a time for the Sections to rise. It ought to be possible for them to begin their work at 9.30, and go on till 1.30. The principal thing to be urged against the earlier time for assembling is the fact that Council meetings are held at 9.30, but that is certainly not an insuperable objection.

Speaking generally, it would appear that better work is done by having only a few specially chosen subjects for discussion than by reading a lot of papers by individual authors.

The Association should furnish each sectional meeting with a requisite number of abstracts of papers, whether they are introductions to discussions or separate contributions. It would be far easier to consider the principal points to be noted in a discussion which is to take place on a previously-arranged topic or on an individual paper if the author had set down in concise form the aspect of the questions with which he proposed more particularly to deal. A resolution bearing on this question was carried unanimously in the Obstetric Section. It is a very important matter. It would facilitate discussion and save the secretaries the thankless task of pestering authors for abstracts of their papers, which they would be more ready to supply than they now are if they knew that a practical use would be made of them at the time of the meeting.

### THE PATHOLOGICAL MUSEUM.

The first view of the Pathological Museum was an astonishing one. Erections of an uncouth shape were piled up for the reception of specimens. The object of getting these on a level with the eye of the observer might surely have been attained without the heavy and inartistic effect which was produced by the means adopted. But the drawings, the specimens, and the microscope slides were so good, that a day in the museum was an education in pathology. The collection was, indeed, a special feature of the meeting, and was visited by a very large number of members. The Engineering Drawing Rooms of University College, which were allotted for the purpose, were admirably adapted for a museum in which so much exhibited was to be seen under the microscope. The light was perfect, and the space allowed almost everything to be placed so that it could be thoroughly well seen. By far the largest number of specimens was obtained from the hospital museums in Bristol, although the collection was enriched by many noteworthy contributions from private collections.

The Bacteriological Exhibition by Drs. Blaxall and Plimmer was so complete that almost every micro-organism was exhibited as a culture, and most of the pathogenic ones were on view under the microscope. Dr. Woodhead exhibited some beautiful preparations of the Plague bacillus from Hong Kong. Mr. Stoddart contributed some very interesting cultures showing the effect of opaque water in preventing the sterilising effects of sunlight. An eminent *savant*, who in his general appearance rather affects the "trick of singularity," found during part of the time that he was in charge of this department that the place was rather hot. He accordingly removed his coat and regaled himself with a cigar. A lady doctor who came into the museum at the time said to him, "You don't look much like a medical man;" to which he replied, "Neither do you."

Two excellent departures were made from the usual pathological museum practice. It is extraordinary that up till now the anatomical, physiological, and pathological collection has always been catalogued with the foods, drugs, &c., of the so-called Annual "Museum." This year a most useful catalogue was printed separately, and for the first time the exhibits were arranged on an anatomical basis instead of, as hitherto, under the names of the exhibitors.

The excellence of the Pathological Museum no doubt took off attention from the Medical School Museum, which is now in admirable order and well adapted for teaching purposes. The visit of the Association certainly had much to do in stimulating some of the professorial staff to bring it to its present state.

Forming an adjunct to the Pathological Museum, there was in a separate room a large loan collection of human crania, together with casts, drawings, and photographs, illustrating the chief races of Britain from the earliest times. About seventy skulls, many of them of great antiquity, were lent by private collectors and local museums. A number of foreign crania and photographs were shown as types of various other races. Some splendid volumes of plates and some diagrams showing the *habitat* of prehistoric races were highly appreciated. A few anthropological instruments from the Cambridge Scientific Instrument Company were also shown. The wish was strongly expressed that a permanent anthropological museum, illustrating the chief racial types, could be formed at the Medical School, since the subject is becoming one of great importance and interest. Local discoveries would not then be so often sent away to distant collections, as is the case now.

### THE ANNUAL MUSEUM.

The Exhibition of Foods, Drugs, Instruments, Books, and Sanitary Appliances was held in the Drill Hall, Queen's Road, and in every way proved a great success. The whole of the space available (150 feet by 90 feet) was secured by exhibitors; and large as was the hall for the purpose, it would not have been difficult to have filled a building one half as large again. This speaks uncommonly well for the energy and enterprise which are brought to bear upon this yearly show. Upwards of one hundred firms were exhibiting; every one had something new or instructive to show, and none of them spared expense in displaying their goods amidst most artistic surroundings, resulting in the production of a general effect that was admired by everybody. The hall was not only commodious, but it was of good shape, it was well lighted, and most important of all it was next door to University College and the Museum and Library in which the sectional meetings were held. This situation of the building ensured a good attendance of visitors. The correspondence received since the Association week shows that the exhibitors are to be congratulated upon their success, especially as this is the recompense hoped for in return for all the time, trouble, and cost which their display necessarily entails. In future they should however bear in mind that Friday afternoon is almost the only time that many of the doctors are free from engagements, and that therefore everything should be left in place till the official time for closing. Much disappointment was expressed by those who went on that afternoon and found that many of the stands were being dismantled. To see even a few untidy places leads to the inference that there is nothing worth seeing, and consequently many visitors go away at once. This is not the place to describe the exhibits in detail, but it is well to call attention to the commendable enterprise shown by the Harrogate Corporation, the Leamington Spa, and the Woodhall Spa in making known to the Medical Association the particular virtues of their respective waters and the natural beauties that are to be found in and around their towns. Clifton in future years should do likewise.

In a room specially arranged for the purpose was a Museum of Antiquities relating to Medicine, which was one of the most interesting things in connection with the whole meeting. The thanks of all were due to Messrs. Oppenheimer for enabling members to see this collection illustrating the practice of our art in ancient times, and which they had been instrumental in bringing from Rome, where it had been arranged for the International Medical Congress last April. Dr. Luigi Sambon was in charge of this treasure-house and very courteously explained its contents, a catalogue of which was given to each visitor. Amongst the things shown were many votive offerings or *donaria* which were hung at the shrines of medical deities or thrown into healing streams. The greater part here were Etruscan and of terra cotta, but a few were of bronze. Many of them represented the diseases for which the cure was invoked, and it was interesting to note that disease of the left ovary seemed then more frequent than that of the right. Some figures depicted the act of parturition in the kneeling position. Other objects were Etruscan feeding-bottles and drop-bottles. There was a large number of instruments taken from the tomb of an oculist of about the fourth century. There was sufficient evidence in this room to show that the surgical practice of the Romans was of a very high order. To see vaginal and rectal specula of the first, second and third centuries which would do credit to the present day, and to see a pictorial representation of the operation of tracheotomy in the ninth century, make one hesitate in referring to the "progress" of surgery in recent years.

### THE WORK AT THE HOSPITALS.

Many members were shown over the Royal Infirmary, the General Hospital, the Children's Hospital, and the Eye Hospital.

Great interest was taken in the Museum of the Infirmary. At this institution the members of the staff each morning showed cases of interest, which included a case of sarcoma of buttock and a case in which the right vagus nerve had been divided during Ballance's operation. Results of operations for radical cure of hernia, of abdominal sections, and of Pearce Gould's operation for cancer of penis were also shown. On Tuesday Mr. Paul Bush operated on a case of strangulated hernia. On Friday morning Mr. Greig Smith, before a large company, performed three operations. One was hysterectomy for a large uterine fibroid; another was nephropexy; the third was removal of a large scirrhus mammæ with extensive infection of axillary glands. In each operation Mr. Greig Smith showed some novel peculiarity of method. The visitors were specially interested in a plan which he adopts in closing denuded surface in mammary excision. Afterwards a visit was made to Mr. Greig Smith's wards, and various cases under treatment were exhibited and discussed. Amongst these were hysterectomy for cancer, nephropexy, several radical cures for hernia, double ligature of the femoral arteries for double femoral aneurism, and enterectomy for cancerous growth in the transverse colon.

On Wednesday, August 1st, by invitation of Mr. Cross, most of the members of the Ophthalmic Section, including several American and foreign oculists, visited the Eye Hospital, and were shown the arrangemeuts for the work done there. Mr. Cross exhibited upwards of sixty patients on whom he had performed the operation for extraction of cataract without iridectomy. The cases were not selected, but comprised those who were able to attend out of all the patients upon whom he had practised simple extraction at the Hospital. The very large majority of eyes showed the perfect result that it is possible to obtain by this method of operating. Some of the others, less successful, showed anterior synechia to a more or less definite degree; but almost all of them had good vision, and were useful and unirritating organs. Mr. Cross invited special examination of the complicated cases, so that criticism might be encouraged and any disadvantages in the operation might be fully exposed.

### THE DAY ENTERTAINMENTS.

*Visits to Factories.*—On each day of the meeting, parties were made up for visiting many of the interesting works in which the trade of Bristol is carried on. In some cases the owners had provided an excellent tea for their visitors, who were also provided with samples of some of the manufactured products.

*Medical Missions Breakfast.*—On Wednesday morning many members of the Association and their ladies were entertained at breakfast in the Lecture Hall of Victoria Chapel by the Church Missionary Society and the Edinburgh Medical

Missionary Society. Dr. E. Long Fox, who was in the chair, made some appropriate observations, and then addresses of great power were given by Dr. E. Neve from Cashmir, and by Dr. Thompson from Hong Kong. The value of medical work in the mission-field cannot be overestimated; and the amount of good work which these gentlemen showed to be possible proves that no one could embark upon a more interesting and useful professional career than under the auspices of one of the various Medical Missionary Societies. At the same time the influence of men so engaged is utilised for teaching of the highest kind.

*Luncheon at Clifton Spa.*—A large number of members of the Association and other guests were entertained on Wednesday at luncheon by Mr. G. Newnes, M.P., whose energy and public-spirited action are doing so much to restore the popularity of Clifton as a health-resort. Of the entertainment it is unnecessary to say more than that the proceedings passed off to the complete satisfaction of all present, and that there was no eager desire for dinner in the evening of that day. After lunch there were a few short speeches, and at a given signal the water was turned on simultaneously by the Mayoress and Mrs. Newnes, who immediately partook of some of the famous Hotwell Spring. The Pump-room in which the luncheon took place is the only completed part of the contemplated Spa buildings. No finer room devoted to a similar purpose exists in England, and it is a great pity that local interests prevented Mr. Newnes from carrying out his original plan of having its magnificent *façade* in the upper roadway. Mr. Newnes said that the water provided in the Pump-room came from the original spring. Effectual measures have been taken to prevent any percolation of sewage into the water, the supply of which is probably about 40 gallons a minute. It is Mr. Newnes's intention to have the whole system of baths and the hydropathic establishment in working order by October, 1895. The arrangement for the external application of the water will, as may be seen from the plans, bear comparison with any baths in Europe. The hydropathic establishment will afford accommodation for fifty visitors.

*The Garden Parties.*—If the weather had only been more favourable the garden parties would have been long remembered among the principal social successes of the meeting.

On Wednesday afternoon Mr. Lewis Fry and Miss Fry invited the members of the Association and a large number of residents in the neighbourhood. The beautiful grounds are in themselves one of the sights of Clifton, containing as they do a magnificent avenue of yew trees, and the ample terrace giving such excellent views of the old city and its docks and the Somersetshire hills beyond. The weather was threatening, but fortunately no rain fell, and the playing of the band of the Clifton Zoological Society was much appreciated. The gardens gave plenty of space for the numerous guests, and the attention of the hostess to every detail rendered the gathering a delightful success.

If the rain kept off on Wednesday, it certainly made up lost time on Thursday, for it came down in torrents and considerably interfered with the enjoyment of the entertainment which Sir Greville and Lady Smyth had provided for a large number of guests at Ashton Court. The glorious drive through the deer park is always a thing of beauty, on account of the fine old trees and the charming views afforded of the city and surrounding country. The host and hostess received every one with the most hospitable cordiality. The attractions in the house were many ; not the least is the excellent zoological collection, which includes many of Sir Greville Smyth's trophies of the chase in many lands. In the grounds, the flower beds of which were a blaze of beauty, several tents were erected, and in these refreshments were provided on a most lavish scale. The band of the Royal Marines played, and the entertainment only lacked sunshine to make it perfect.

Also on Thursday afternoon the Head-master of the Grammar School and Mrs. Leighton gave a garden party to which a large number of persons went. The spacious building and ample grounds were unreservedly placed at the disposal of the guests. The out-door portion of the entertainment was sadly marred by the heavy rain, and everyone was obliged to remain in the school buildings. Mr. George Riseley played the organ; and with a party of glee singers, and the band of 1st V.B. Gloucestershire Regiment, the guests were well entertained. Refreshments were provided on a most liberal scale.

On Friday Dr. Eager and Mr. and Mrs. Seymour gave a garden party at Northwoods. The distance from Bristol rendered the number of guests smaller than it would otherwise have been.

*Medical Temperance Breakfast.*—This took place at the Royal Hotel on Thursday, and was given by the invitation of the National Temperance League. Dr. E. Long Fox presided. Very interesting addresses were given, amongst them being one by Dr. Annie Cornall, who is just starting for work in India. The interest in these breakfasts has grown year by year since they were instituted twenty-five years ago, and the increased numbers present, with the remarks of the various speakers, bore testimony to their utility in bringing the subject of temperance before the profession.

*Luncheon at the Merchant Venturers' Hall.*—By invitation of the Master of the Society of Merchant Venturers (Mr. R. A. Fox) about eighty medical men lunched on Friday in the fine hall of the Society. Words cannot adequately express the special excellence of this entertainment, which was a delight to the eye as well as to the palate. It was decidedly a triumph of fine art in table decoration and in cookery, and the wines were well chosen to accompany a meal which a Roman Emperor might have envied. The cigar which followed was a worthy sequel. Those who missed this function lost one of the best things of the week.

To provide for various tastes other attractions were announced, amongst which were an Organ Recital at the Colston Hall on Wednesday; an inspection of "Old Bristol" on Wednesday and Friday; and an "At Home" by the Mayoress of Bristol at the Mansion House on Friday, with the City plate and a collection of old china on view.

### THE EVENING ENTERTAINMENTS.

*Conversazione by Museum Exhibitors.*—The social capabilities of the large hall in which the exhibition was held were fully taken advantage of on Tuesday in the evening of the opening day, when the exhibitors gave a conversazione to the visiting doctors and their ladies and some other guests, in number altogether over a thousand. A most excellent programme of music was given by the band of the Royal Marine Artillery, Gosport. The spacious avenues between the lines of exhibits afforded ample space for the movements of the large gathering of people. In the course of the evening Prof. Philipson, the past president, in the absence of Dr. Long Fox, who was detained at the general meeting, formally declared the exhibition open, and thanked the exhibitors for their entertainment, and for all the trouble and anxiety to which they had been put in giving the members of the Association the opportunity of seeing the latest additions to pharmacy and the armamentarium of surgical art. Mr. Charles Townsend, M.P., replied on behalf of the exhibitors. Refreshments were served in the long corridor leading to the hall, and from the crowd which was always present it may be inferred that some of the guests never got any further than the specimens of food and stimulant which were to be found there. This conversazione, arranged to follow directly after the President's address, was a new departure in the festivities of the Association week, and formed a most pleasant assembly at a time for which nothing had been hitherto devised.

*President's Reception.*—Nearly 2,000 guests were present at this remarkably brilliant *fête*, held at Clifton College. The visitors, among whom were many of the residents of the neighbourhood, were received by Dr. and Mrs. Edward Long Fox in the Council Room. In Big School, the concert given by the Bristol Orpheus Glee Society, conducted by Mr. George Riseley, was worthy of its national fame and was an immense pleasure to those comparative few who were able to get into the room. A pleasant feature in connection with this entertainment was the easy access to the Zoological Gardens, the use of which was obtained for the guests for the evening. The effect of the illumination of these charming gardens was excellent, and the display of fireworks was good. A set-piece represented "The Good Samaritan": in this, either by accident or as a graceful compliment, the patient lasted longest. After the return to the College there was an organ recital by Mr. Riseley. Supper was served in a large tent provided for the purpose in the College grounds. Assemblies of which out-door attractions form a part would, if the weather could be relied upon, furnish much the most enjoyable and manageable way of entertaining the Association, the numbers of which are now rather unwieldy for any rooms except those of the largest size.

*Public Dinner*.—This was held in the large Victoria Room. Places were laid for 270, and nearly that number was present. Dr. Fox presided. The room was admirably decorated, the seats were well arranged, but the dinner was less sumptuous than the extremely elegant *menu* card led the guests to expect. The card was a three-page one, decorated front and back with symbolical designs specially produced. The design of the centre panel of the front was named "A Consultation with Æsculapius." On other parts of the card were sketches and devices of local heraldic and medical interest. The central panel of the back contained an appropriate description of the occasion and an emblematic device giving the dates of the three Bristol visits of the Association. The card was printed in a dark reddish-brown on vellum, and attached to it by means of terra-cotta silk was a handsome wax seal representing the Civic Arms. The wine was excellent, and perhaps enough was served for the welfare of the guests, but some of them had a great difficulty in getting what would be deemed even a moderate supply. Bristol milk was provided, but it was served (Shades of Pepys and Macaulay, hear it not!)[1]—in a liqueur glass! If Father Mathew could have been present at the 1863 and 1894 dinners (both of which were presided over by men of the strictest temperance), his warm Irish heart would have been much gladdened by seeing the progress his principles had made. As usual at these feasts, conversation was rendered difficult by

[1] On June 13th, 1668, Pepys was in Bristol and formed one of a party that had "good entertainment of strawberries, a whole venison-pasty cold, and plenty of brave wine, and above all Bristol milk." Macaulay, writing on the state of England in 1685, alludes to Pepys's visit to Bristol, and notes the hospitality of the city, "especially the collations with which the sugar refiners regaled their visitors. The repast was dressed in the furnace, and was accompanied by a rich beverage made of the best Spanish wine, and celebrated over the whole kingdom as Bristol milk."—*Hist. of Eng.*, vol. i., ch. iii.

the noise of the band. The speeches were very imperfectly heard by many of the audience, probably owing to the fact that few people know how to use their voices in a large room.

*Smoking Concert.*—In accordance with what has now become an annual custom, the exhibitors in the Museum gave a smoking concert on the night of the public dinner, and invited the members of the Association to it. The entertainment, of which Dr. D. S. Davies was the chairman, was held in the Alexandra Hall, which was packed to the doors. A long programme of music occupied the guests till a late hour.

*Mayor's Concert and Ball.*—When the Mayor was informed that the Association would visit Bristol this year, he consulted some of the more influential of his fellow-citizens, with the result that it was determined that the British Medical Association should be entertained in a manner worthy of the traditions of the ancient city. A committee was formed, consisting of the Mayor, the High Sheriff, the Master of the Society of Merchant Venturers, and many others. The famous Bristol Madrigal Society consented to give one of its enjoyable concerts. Colston Hall, which at ordinary times is sombre and bare, was, under the direction of a committee of which Mr. Lewis Danger was the active chairman, transformed into a handsome drawing-room, the centre of which was at first arranged for a concert, to be altered a little later, as if by magic, into a most perfect ballroom. The guests, who in the course of the vening must have numbered about 1,800, were received by the Mayor and Mayoress, and the concert was at once given by the Madrigal Society, under the leadership of Mr. Daniel Rootham ; their performance was, as usual, exquisite, and gave intense delight, especially to those who came from a distance and who had never had an opportunity of hearing such renderings. After the concert the chairs were quickly moved away, while the assembly partook of light refreshments served in the Arch Room. The ball afforded a more than usually charming spectacle of its kind, due largely to the tasteful character of the decorations, the abundance of room, and the brilliant dresses of the company, among whom were many officers of the regular and Volunteer forces. At eleven o'clock a splendid supper, with wines of fine quality, was served in the small hall at three tables, each accommodating one hundred guests. The arrangements of this were admirable, and were carried out by a committee of which Mr. Charles Wasbrough was the indefatigable chairman. Altogether this was one of the most successful entertainments ever given in the city. From first to last everything went smoothly, and all was done without ostentation and without stint. Everyone was delighted, and the general opinion was that nothing could have been better. Among the many noteworthy functions of the week, this stands out pre-eminently as the most brilliant of all.

THE SHORT EXCURSIONS.

*The Docks.*—On Thursday, by the invitation of the Docks Committee, a party went in special steamer round the docks and up the river to Hanham. This was a great attraction, and many in excess of the one hundred to which the number was limited applied for tickets. Unfortunately, the weather was bad. The excursion, however, was greatly enjoyed by the visitors, who were sumptuously entertained at lunch.

*Bath.*—The Mayor and Corporation of Bath invited two hundred members to luncheon on Friday. A special train conveyed the party from Clifton Down at 1.30. The luncheon, presided over by the Mayor, left nothing to be desired, and a most enjoyable time was spent till 3.30, when the guests were taken to the Pump-room, and afterwards shown the baths. Another special train, which left Clifton Down at 3.20, brought some additional visitors, who with the other party were received in the Pump-room by the Bath doctors, and entertained by them in a most handsome way. The return journey was made at 6.18.

*Clevedon.*—Forty persons accepted the invitation of the Clevedon doctors and residents to visit this charming watering-place on Friday. A special train at 12.30 took the visitors to Clevedon, where they were met by the resident doctors, and then driven to the Walton Park Hotel, where they were received by Mr. Charles Hill, the Chairman of the local Committee. After an excellent luncheon had been served carriages took the party to see the extensive views of the neighbourhood. A visit was then paid to Clevedon Court, the residence of Sir Edmund Elton, who was unavoidably absent. The house and grounds and the famous Elton Pottery much interested the visitors, who, after a drive through Clevedon itself, proceeded to Mr. Charles Hill's, where they were hospitably entertained at tea. The return special train arrived in Bristol at 7. This was a most enjoyable afternoon. The weather was splendid.

*The Water Works.*—The Friday visit to the Bristol Water Works was much appreciated by those who accompanied Mr. A. J. Alexander, the Secretary of the Company, on the delightful drive to the storage reservoirs and filter-beds at Barrow. Tea was provided by the liberality of the Company. Mr. Alexander gave an account of the rise and development of the Bristol Water Works system. The water is derived chiefly from springs in the carboniferous limestone and the conglomerate of the Mendip Hills, and from the deep wells in the new red sandstone. The storage capacity at Barrow is 850,000,000 gallons. Extensive works are now in progress in the valley of the Yeo, where a reservoir is in course of construction with a holding capacity of 2,000,000,000 gallons. The daily delivery per head to the population is 22 gallons.

### THE LONG EXCURSIONS.

*Berkeley and Thornbury.*—This was the most popular of the day excursions, which all took place on Saturday. The full number of one hundred tickets was quickly disposed of, and the party left Bristol in carriages at 9.15. The view of the Severn from Almondsbury Hill and the drive through Lord Ducie's park at Tortworth were much enjoyed. Upon arrival at Berkeley the visitors were received by Lord and Lady Fitzhardinge, who entertained them at a splendid luncheon, served in the banqueting hall. Afterwards the company was divided into two groups, one conducted by his lordship and the other by his steward, and a thorough inspection was made of the Castle, including its chapel and the dungeon in which Edward II. was imprisoned previous to his brutal murder. One of the attendants handled a rapier, with the remark: "This was the thing as did it." Several interesting things associated with Jenner were also seen. The carriages were again in requisition, and took the party to Thornbury Castle, where they were received and entertained at a very acceptable tea by Mr. Stafford Howard and Lady Rachel Howard, the former of whom showed his guests round the Castle. The Church, the details of which were explained by the Rector, was also visited. The return journey was resumed shortly before 7, and Clifton was reached at 8.30, after a day of great pleasure with which the afternoon rain but slightly interfered.

*Glastonbury and Wells.*—Those who had chosen this excursion were taken to Glastonbury in a special train, which left Bristol at 9.25. Carriages were in waiting, and in a short time the Lake village was reached. Excavations are still in progress, under the direction of Mr. Arthur Bulleid, who discovered it about two years ago, and who was present on this occasion to explain its details. Glastonbury was reached in a short time, and the Abbey was at once visited by the party, who were fortunate to have Mr. J. G. L. Bulleid for their guide. A comparatively short time could be spent at it, as it was necessary to reach Wells about two, where luncheon was served at the Swan Hotel. Some of the members attended service at the Cathedral at three, and others visited the gardens of the Bishop's Palace. Shortly before four, Canon Buckle took the whole party over the Cathedral, and described its details with great lucidity. He also took them to the Vicars' Close, and at five to the Deanery. The Dean and his ladies were unremitting in their attention to their guests at tea. Some of the party took tea with Dr. Fairbanks at his house. After a most enjoyable day, in which there was very little rain, the party, which numbered sixty-five, left Wells in the special train at 6.5.

*Weston-super-Mare and Cheddar.*—About forty members visited Weston-super-Mare, by invitation of the local members of the Association. They left Cumberland Basin at 9.30, in the steamer *Ravenswood*, which had been specially chartered for this and the Ilfracombe trip. Upon reaching Weston pier about 11.15, they were met by the local Medical Committee. Carriages were provided for the visitors, who were taken to the Ancient British encampment on Worlebury Hill, a full description of which was given by one of the party. After this a visit was paid to the Grove Park and other popular places of resort in the town. An excellent luncheon was served at the Grand Atlantic Hotel. About three the visitors returned to their carriages and were driven to Cheddar. Just after leaving Weston it began to rain heavily, and continued to do so during the whole drive. The party arrived at Cheddar at 5.45, and were received at the residence of Mr. Statham, by whom and Mr. Openshaw they were entertained at tea. The visitors had only about ten minutes in which to visit the Cheddar caves, as they had to return to Bristol in the special train which brought the medical party from Wells. The Vicar of Cheddar, who was present, had arranged for a visit to the Parish Church, but the time would not allow this to be accomplished.

*Ilfracombe.*—A channel trip is always enjoyable to those not overtaken by the sorrow of the sea, and the members of the Association and their friends who elected to take the voyage to Ilfracombe were rewarded by better weather and a calmer sea than the preceding days had led them to expect. They were accompanied for the first part of the journey by those who had accepted the hospitality of the doctors of Weston-super-Mare. After Weston had been left, the party thoroughly enjoyed the view of the varying coast line, with the rolling meadow land, the heights of Porlock Bay, and the rugged headlands of the Devon coast. The sun, though somewhat coy, shone fitfully, and the outward voyage was made under very favourable conditions. Most of the passengers had found their sea-legs by the time Lynmouth was reached, and they were able to do full justice to the excellent lunch provided on board. Some of the more daring risked the boat landing, and deserted to explore Lynmouth and Lynton. Ilfracombe was reached about 2 o'clock; about an hour was available for enjoying the scenery of this delightful resort. Though a sea fog somewhat marred the full pleasure of the homeward voyage, it was not enough to drive the voyagers below. A substantial tea was served at 5 o'clock, and the party arrived at

Bristol between 7 and 8, all the better for a sea-breeze after the exacting functions of the week.

*Chepstow, Tintern, and Symonds Yat.*—The party visiting these charming places numbered about eighty. They proceeded from Bristol in a special train at 10.15, and arrived *viâ* Severn Tunnel at Chepstow, where Dr. Yeats took them over the ruins of Chepstow Castle. Carriages then took them through Piercefield Park to the Wyndcliff, where Dr. Yeats described the scenery and the geology of the neighbourhood. Having descended to the Moss Cottage, the party drove to Tintern, where luncheon was provided in a tent erected by permission of Mr. Baldwin in the Abbey Grounds. By his attention and thoughtfulness, Mr. E. P. King added much to the enjoyment of the party both at Chepstow and Tintern. After luncheon, Dr. Yeats conducted the party through the awe-inspiring remains of the Abbey, which he fully described. The special train conveyed the visitors to Symonds Yat, where they were met by Mr. Edward Turner and Mr. Henry Southall. Tea was served, and then, under the guidance of these gentlemen, the party went to the top of the precipitous headland, from which a magnificent panorama is obtained of the lovely Wye scenery. Mr. Southall gave an account of the antiquarian and botanical interest of the district. The party left Symonds Yat at 7.20, and came back by way of Pontypool Road and Newport to Bristol at 9.37, having had a thoroughly satisfactory day.

The pleasure of the excursions was considerably increased by the kindness of many persons who presented handbooks and guides in sufficient number for each member of the excursion parties to receive a copy. Mr. Baker's *Guide* contained a great deal of interesting information about the Bristol Channel Circuit. The Bristol Executive gave small guides to Bath, Clevedon, Weston-super-Mare, and Tintern Abbey. Mrs. George sent a *Guide to Wells*, Mr. Hancocke Wathen had a paper on Glastonbury and Wells printed, which Mr. Robert Hall Warren had read at a meeting of the Social Book Society. Messrs. P. and A. Campbell gave each of the passengers on the *Ravenswood* one of their *Bristol Channel Guides*. Dr. Yeats wrote and had printed for the occasion an account of Chepstow Castle and Tintern Abbey.

Enjoyable as these excursions were, they would have been still more so if each person joining in them had been supplied with the names and addresses of those who constituted the party. In large parties such as these usually are there are many who, if the names were known, would gladly take the opportunity of making acquaintance with one another. On future occasions lists of the several parties should be printed on Friday, and a copy given to each member on starting for the excursion.

### GENERAL SUMMARY.

If we are to judge by the universal expression of satisfaction and pleasure heard on all sides, the 1894 Meeting of the British Medical Association must be pronounced a decided success. A distinguished foreigner, on the eve of his departure, wrote this note, which is only a sample of the sentiment that was expressed in various ways:—

> Ladies and Gentlemen,—Allow me to return hearty and sincere thanks of guests of this illustrious Society! English hospitality is well known in the whole world. This hospitality favours us, rejoices us, and cheers us; and the opportunity to join the celebrated discussion on science honours us in a very high degree. Having got a very honourable reception of our professional brethren, we are also received in a most amiable and kind manner by the citizens of this noble, mighty virtuous, and industrious city, as well by the gentlemen as—I am proud to say—by the ladies. Here we see distinctly in what high degree progress of science is afforded by wealth, and science favours health and wealth. Returning home, we are fully convinced that Horace was throughout mistaken when he talked of "Britannos hospitibus feros." If this old Roman could have come to Bristol, he would have said, "I call again, Ladies and Gentlemen!"

The scientific part resulted in much sound work of real practical benefit. During the week hospitality, public and private, was profuse, and through an inability to be in two places at once there must have been many fond regrets for opportunities missed. In connection with the development since 1863 of the social part of the meeting, a statement of the *Bristol Times* of August 8th of that year is of interest. In the course of an editorial it said: "That the medical sages know how to be merry as well as wise, the fact that they opened their proceedings with a popular entertainment at the Institution and closed with a good dinner at the Victoria Rooms, may be taken as evidence."

The hearty manner in which all the medical practitioners of the city and neighbourhood took upon themselves the duty of making things pleasant for the guests added much to the success of the meeting, and this, with the kindness and liberality of public bodies, worthily kept up the record of the hospitality of Bristol. The meeting will be remembered for the earnest endeavour of all present to prove that union is strength, that personal good-fellowship and a sense of the honourable nature of our aims are bonds that bind us together for all good work, and that in quiet self-devotion and earnest strivings after truth the profession in the end of the 19th century is worthily following in the steps of its great medical ancestors.

Now that it is all over, the prevailing thought is one of thankfulness, and for two reasons: in the first place, that our visitors profited scientifically and undoubtedly enjoyed themselves; and in the second place, that in all human probability we shall not see them again collectively for another 30 years.

### List of Officers and Members of Committees.

Dr. E. LONG FOX, *President;* Dr. W. JOHNSTONE FYFFE, *Treasurer;* Dr. E. MARKHAM SKERRITT, *Hon. Local Secretary.*

*These were members of all Committees.*

FINANCE COMMITTEE.

Dr. W. Johnstone Fyffe, *Chairman;* Dr. Arthur B. Prowse, *Secretary;* Dr. A. J. Harrison, Mr. Augustin Prichard, Mr. J. Greig Smith, Dr. J G. Swayne, Mr. S. H. Swayne.

PRINTING AND PUBLISHING COMMITTEE.

Mr. G. Munro Smith, *Chairman;* Dr. R. G. P. Lansdown, *Secretary;* Mr. J. Dacre, Mr. L. M. Griffiths, Dr. G. Parker, Mr. C. K. Rudge, Dr. James Swain, Mr. J. H. Wathen, Dr. P. Watson Williams.

### RECEPTION COMMITTEE.

Dr. A. J. Harrison, *Chairman;* Mr. James Taylor, *Secretary;* Mr. W. R. Ackland, Dr. Barclay J. Baron, Mr. J. Paul Bush, Dr. J. Michell Clarke, Dr. B. B. Fox, Mr. C. F. Pickering, Mr. J. Stewart.

### GENERAL PURPOSES COMMITTEE.

Mr. Arthur W. Prichard, *Chairman;* Dr. Bertram M. H. Rogers, *Secretary;* Mr. W. M. Barclay, Dr. G. H. Barker, Mr. H. F. Devis, Mr. G. Fendick, Mr. A. N. Godby Gibbs, Mr. F. T. B. Logan, Mr. C. E. Matthews, Mr. H. Ormerod, Dr. W. E. Pountney, Dr. H. Waldo.

### PATHOLOGICAL MUSEUM COMMITTEE.

Dr. R. Shingleton Smith, *Chairman;* Mr. C. A. Morton, *Secretary;* Dr. Barclay J. Baron, Dr. J. Michell Clarke, Mr. F. R. Cross, Mr. J. Dacre, Dr. D. S. Davies, Dr. W. Dowson, Dr. F. H. Edgeworth, Dr. T. Fisher, Dr. W. J. Fyffe, Dr. A. J. Harrison, Dr. A. E. Aust Lawrence, Mr. H. F. Mole, Dr. G. Parker, Dr. J. J. Powell, Dr. B. M. H. Rogers, Dr. W. B. Roué, Dr. J. E. Shaw, Mr. G. Munro Smith, Dr. James Swain, Mr. J. Taylor, Mr. J. H. Wathen, Dr. P. Watson Williams.

### LOCAL ENTERTAINMENTS COMMITTEE.

Mr. F. R. Cross, *Chairman;* Dr. W. H. C. Newnham, *Secretary;* Dr. T. J. Colman, Mr. Hedley Hill, Dr. W. T. Maddison, Mr. J. H. Wathen, Mr. J. B. Webb, Dr. C. A. Wigan.

### DINNER AND LUNCHEON COMMITTEE.

Dr. H. Marshall, *Chairman;* Mr. J. Paul Bush, *Secretary;* Mr. A. F. Blagg, Mr. F. R. Cross.

### EXCURSIONS COMMITTEE.

Dr. D. S. Davies, *Chairman;* Mr. J. Hancocke Wathen, *Secretary;* Mr. A. F. Blagg, Dr. E. Crossman, Dr. R. Eager, Dr. W. Fairbanks, Mr. W. G. Grace, Mr. L. M. Griffiths, Dr. W. J. K. Millard, Dr. G. F. Rossiter, Dr. R. Roxburgh.

### ANNUAL MUSEUM COMMITTEE.

Mr. L. M. Griffiths, *Chairman;* Dr. J. E. Shaw, *Vice-Chairman;* Mr. John Dacre, *Secretary;* Mr. W. M. Barclay, Dr. D. S. Davies, Mr. Hedley Hill, Mr. H. F. Mole, Dr. G. Parker, Mr. W. J. Penny, Mr. C. F. Pickering, Mr. E. J. Sheppard.

### EXECUTIVE COMMITTEE.

The Executive Committee consisted of the local Members of the Management Committee (see page 174), and of the Chairman and Secretary of each of the other Committees.

### LOCAL EDITOR OF DAILY JOURNAL.

Dr. P. Watson Williams.

